# Severe Ethanol Poisoning Among United States College Fraternity Pledges

**DOI:** 10.7759/cureus.58650

**Published:** 2024-04-20

**Authors:** Todd S Ing, Armen Sam, Hon-Lok Tang, Keith K Lau

**Affiliations:** 1 Department of Medicine, Stritch School of Medicine, Loyola University Chicago, Maywood, USA; 2 Department of Medicine, Loyola University Medical Center, Maywood, USA; 3 Department of Medicine and Geriatrics, Princess Margaret Hospital, Lai Chi Kok, HKG; 4 Department of Pediatrics, The Chinese University of Hong Kong, Shenzhen (CUHK-Shenzhen), Shenzhen, CHN

**Keywords:** hemodialysis, poisoning, ethanol, hazing, pledgers

## Abstract

Hazing is a longstanding tradition in university and college fraternities. This practice often uses alcohol as a penalty during hazing rituals, resulting in severe ethanol poisoning and even death among pledges. Typically, the serum ethanol levels in these poisoned students are extremely high. Preventing severe ethanol poisoning is crucial, and can be achieved through education about the harms of these hazing activities. Hemodialysis is an effective treatment for severe ethanol poisoning as it removes the excess alcohol in a timely manner.

## Introduction and background

"No, I haven’t any particular fears for you-only those that trouble any father whose son or daughter reaches the age when it is hard to stay out of the way of a potent chemical called alcohol [1]." Ethanol is a small, water-soluble molecule that is not protein-bound, and can, hence, easily traverse biological membranes. Its molecular weight is 46.07 g/mol, and the molecular formula is CH3CH2OH. When alcohol is consumed, it is distributed in the total body water [2]. The first step in the metabolism of ethanol is its conversion into acetaldehyde by the enzyme alcohol dehydrogenase. The subsequent steps involve the conversion of acetaldehyde into acetate, then to acetyl CoA, and finally into CO2 and H2O [2]. Ethanol is naturally eliminated from the body through alcohol dehydrogenase in the liver. Factors affecting the elimination rate of ethanol include age, history of ethanol use, and liver function [2]. On average, 12 oz (1 oz = 30 mL) of beer and 4 oz of wine are approximately equivalent in raising the blood alcohol concentration (BAC) by 25 mg/dL (5.4 mmol/L). A toxic dose of ethanol is 5 g/kg in adults and 3 g/kg in children [2]. Children can also have fatal complications at BAC levels as low as 50 mg/dL (10.9 mmol/L) [2]. According to the Secretary of State of Illinois, "Alcohol is the single greatest factor in fatal vehicle crashes" [3]. A BAC of 0.25 percent means that there are 0.25 g/dL (i.e., 0.25 g (250 mg) of alcohol in 100 mL of blood). In Illinois, if a driver is arrested with a BAC of 0.08 percent (i.e., 0.08 g/dL (80 mg) of alcohol in 100 mL of blood), that individual's driving privileges will be suspended for at least six months. The alcohol elimination rate for adults is 20 mg/dL/hr (4.3 mmol/L/hr), and for children, it is 20-30 mg/dL/hr (4.3-6.5 mmol/L/hr) [2].

Ethanol elimination is a zero-order process, which means that the reaction proceeds at the same rate regardless of the concentration of the reactants [4]. At very high BAC, elimination may shift to first order. A first-order reaction occurs when a drug is eliminated at a constant proportion, meaning that if the concentration of the drug is higher, more of the drug is removed. A BAC above 80 mg/dL (17.4 mmol/L) defines legal intoxication in most states in the United States. The average rate of elimination varies among different types of drinkers. The rate for nondrinkers is 12 mg/dL (2.6 mmol/L) per hour, for social drinkers it is 15 mg/dL (3.3 mmol/L) per hour, and for long-term drinkers, it is 30 mg/dL (6.5 mmol/L) per hour [4]. This supports the statement that the rate of elimination increases with more frequent drinking [4]. Contrary to common belief, activated charcoal does not remove ethanol. Currently, the treatment for alcohol intoxication includes the administration of fluids, multivitamins, thiamine, folate, and glucose. Since ethanol is a small molecule, hemodialysis can easily remove it, preventing further organ toxicity in severely ill patients [5].

## Review

Hazing in colleges

Hazing is a ritual practiced in universities and other institutions where new members of a club or society are degraded, humiliated, or abused. Some might think that hazing is a long-gone tradition; however, as recently as 2017, at one of the most popular colleges in America, Louisiana State University (LSU), an 18-year-old died from a hazing accident. Freshman Max Gruver was pledging to join Phi Delta Theta and was at one of the fraternity’s events called 'Bible Study.' This event involved testing a pledge on the Greek alphabet and fraternity history. If the pledge answered incorrectly, he was required to chug a 190-proof (95% alcohol (for the purpose of the present communication, alcohol and ethanol are used synonymously)) drink. Authorities described it as a hazing ritual in which teenage pledges were pressured to chug several bottles of 'Diesel,' a 190-proof grain liquor that federal prosecutors called the 'most potent alcohol on the market by far' [6]. Gruver was left alone from 3 to 9 AM during which he vomited and aspirated pieces of spaghetti, blocking his airway. He was taken to a hospital in extreme illness and later died. Prior to death, his serum alcohol level was found to be 0.495 g/dL (107.42 mmol/L), more than six times the commonly recognized legal limit of 0.08 g/dL (17.36 mmol/L). As a result, Gruver’s parents sued the State of Louisiana (as LSU is a state-run university) and started The Max Gruver Foundation. The foundation aims to expose the evils of hazing by educating high schools, universities, fraternities, sororities, and other student organizations, and it strives to create laws to ban hazing in all colleges. According to Fox News Network, as of March 13, 2023, the Gruver family was awarded 6.1 million US dollars by the State of Louisiana in a wrongful death suit [7]. A former LSU student was found guilty of negligent homicide in causing Gruver’s death and was sentenced to five years in prison, 1,000 hours of community service, a $1,000 fine, and three years of probation [8].

Another hazing-related death from ethanol poisoning occurred at Bowling Green State University. A sophomore, Stone Foltz, 20, died of 'fatal ethanol intoxication' in 2021 after being forced to drink an entire handle of whiskey during a fraternity induction ritual. His parents were awarded 3 million US dollars from the university. With this settlement money, they launched the iamstonefoltz Foundation to support their efforts to end hazing. Two of Stone’s former fraternity members were charged with manslaughter and reckless homicide. One received 42 days of jail time and 100 hours of community service, while the other was sentenced to 42 days in jail and 28 days of house arrest [9].

Two other similar hazing-related deaths involved a University of California, Irvine freshman, an 18-year-old Filipino American in 2019 [10], and a Virginia Commonwealth University freshman, a 19-year-old student in 2021. Both died from ethanol intoxication after drinking large amounts of alcohol as part of fraternity hazing [11].

In order to join a college fraternity or sorority, often a pledge must perform a tradition or ritual known as hazing. Once a pledge has undergone the hazing process and successfully joins the group, there may be other mandatory hurdles to overcome. To secure the right to remain in the group, a pledge may need to maintain a certain grade point average (GPA) in his or her studies, raise money for charity, and/or participate in community service. However, the main, anchoring 'attraction' of many of these pledging gatherings is often characterized by a pattern of vigorous drinking.

On September 1, 2019, the North American Interfraternity Conference (NIC) banned fraternities and sororities from serving hard liquor (anything above 15% alcohol by volume) at their parties across the United States [12]. Despite these NIC bans, however, many fraternities seem to have ignored them and persisted in embracing this hazardous hazing practice, which can lead to alcohol poisoning and resultant death.

Drinking a large quantity of alcohol in a short time can lead to alcohol poisoning [13]. Having extremely high levels of alcohol in one’s system can cause essential areas of the brain, such as the medulla oblongata (in animal models), which controls breathing, body temperature, and heart rate, to malfunction [14]. When these untoward effects manifest, permanent brain damage or potential death can be the unfortunate consequences.

There are more than 2,200 alcohol poisoning-related deaths in the US each year [13]. Statistics show that most deaths occur among people aged 35-64, are more common in men, and most frequently among whites. Alcoholism (alcohol dependence) factors into 30% of all alcohol poisoning deaths. Alcohol overdose results from consuming a large amount of alcohol too quickly.

Alcohol poisoning occurs when the serum (alcohol is measured in serum, not in blood) alcohol concentration (BAC) is above 0.25 percent. A BAC of 0.25 percent means that there are 0.25 g/dL (54.3 mmol/L, MW of ethanol = 46 daltons) (i.e., 250 mg of alcohol in 100 mL of serum). One study states that 1,519 college-age adults between the ages of 18 and 24 die each year from alcohol-related injuries [15]. Most of these injuries result from driving while intoxicated.

Some dire signs/symptoms of an alcohol overdose include vomiting, seizures, and dulled responses such as a lack of a gag reflex, hypothermia, bradycardia, difficulty remaining conscious, confusion, slow breathing, and/or irregular breathing [2]. In the event of severe poisoning manifestations, one should call emergency services immediately to prevent causing further harm and not wait for the appearance of more symptoms. Additionally, the helper should not act like a physician and advise wrongful steps such as cold showers, hot coffee, or walking [16]. These maneuvers will not reverse the effects of the overdose and can actually worsen the situation. While waiting for medical help, one can [17]: (1) Make sure to record accurate information about what and how much alcohol the person has consumed, and any health information such as medications that the patient has been taking, existing health conditions, and allergies to medications; (2) Ensure that the affected person is not left alone, as he or she might get up and fall or choke; (3) Ensure that the patient is in a sitting position or lying on the ground; and (4) If the person is vomiting, ensure that choking does not occur. If the patient is unconscious, roll him or her to one side to avoid aspiration.

As the Father of Modern Medicine, Hippocrates, 460-355 BCE, earnestly advised: 'First, do no harm.' From 2010 to 2017, at least 17 college students are known to have died due to alcohol-related hazing [18]. These unfortunate pledges were from university-sanctioned fraternities or underground fraternities. These deaths resulted from excessive alcohol consumption along with accompanying isolation, humiliation, sex acts, and sleep deprivation. In one case, a student died from falling from a balcony while heavily intoxicated.

Fraternity hazing in the 21st century is in its most appalling state ever, and only a small fraction of the hazing activities have been highlighted in the media. Ninety-five percent of students who have participated in hazing do not report it [18]. According to the Novak Institute on Hazing at the University of Kentucky, 37% of students stated they did not want to report hazing as they did not want to get the group in trouble, 42% stated that other members would exclude them from their parties and activities if a report were presented to the authorities, and 25% of students stated they did not report hazing as they thought their advisers and coaches were already aware of what was going on [18].

Prevention of severe ethanol poisoning

Prevention is better than cure. The life-threatening risks of severe ethanol poisoning should be more widely known by the general public. Although the dangers of excessive drinking are frequently discussed, cases of ethanol poisoning remain rampant. Despite this, intensive and extensive educational efforts by noble organizations, such as "Mothers Against Drunk Driving (MADD)" and "The Alcohol Pharmacology Education Partnership (APEP)," to discourage excessive drinking are still in full and tireless swing. These valuable and selfless efforts deserve our sincere gratitude [19, 20].

Treatment of severe ethanol poisoning

With severe ethanol poisoning, often associated with hypotension, respiratory failure, aspiration risks, unconsciousness, and other life-threatening side effects, ICU management is mandatory. Once first responders enter the victim's home, they should also look for empty alcohol bottles to confirm possible drinking. In addition, the first responders should check the victim’s serum glucose levels, as these can be low with alcohol poisoning [2, 21]. When the body is too occupied to dispose of the excess alcohol, its liver produces less glucose [22]. With the assistance of intensive care, many centers prefer to rely on the body's own metabolic processes to eliminate the excess ethanol [5]. However, in cases of severe poisoning (with serum ethanol levels of 0.45 g/dL (97.7 mmol/L) or higher) [20], an alternative means of disposing of the ethanol might be necessary. Hemodialysis has been applied to patients with severe ethanol poisoning and has proven to be highly effective in promptly reducing serum ethanol levels, along with the satisfactory abatement of associated poisoning symptoms (Table 1) [5, 23-27]. Ethanol’s small molecular weight of 46 daltons (smaller than urea's 60 daltons) and its lack of protein binding [28, 29] make it eminently dialyzable. Hemodialysis can shorten the duration of coma and is expected to reduce the chances of death from profound hypotension, arrhythmias, respiratory depression, and asphyxiation due to aspiration of vomit. However, whether severe cases of ethanol poisoning should generally be treated with this form of dialytic therapy is still under discussion by medical poisoning experts.

**Table 1:** Summary of some patients described in the literature who were treated for alcohol poisoning with hemodialysis. Reference 5, Driscoll D et al.; reproduced with permission from publisher Elsevier. HD: Hemodialysis.

Report	Total Hours of HD	Serum Ethanol, mg/dL, Pre-HD	Serum Ethanol, mg/dL, Post-HD
Wildenauer R et al. [26]	4	615	214
Atassi WA et al. [23]	4	523	100
Elliott RW and Hunter PR [27]	3	465	255
Morgan DL et al. [25]	3.5	407	84
Quispe Gonzales JO et al. [24]	3	650	373
Driscoll D et al. [5]	4	428	204

​​​

## Conclusions

Alcohol-related hazing can lead to severe ethanol poisoning and, at times, death among college fraternity pledges. Such hazing practices should be discouraged. Educating about the harms of alcohol-associated hazing is crucial. Hemodialysis is a promising treatment for severe ethanol intoxication, as it promptly removes the excess poison from the body.
